# Quantitative measurement of internal quality of carrots using hyperspectral imaging and multivariate analysis

**DOI:** 10.1038/s41598-024-59151-y

**Published:** 2024-04-12

**Authors:** Arcel Mutombo Mulowayi, Zhen Hui Shen, Witness Joseph Nyimbo, Zhi Feng Di, Nyumah Fallah, Shu He Zheng

**Affiliations:** 1 College of Mechanical and Electrical Engineering, Fujian Agriculture and Forestry University, Fuzhou, 350002 China; 2 Engineering College, Fujian Jiangxia University, Fuzhou, 350108 China; 3 Fujian Provincial Key Laboratory of Agro-Ecological Processing and Safety Monitoring, College of Life Sciences, Agriculture and Forestry University, Fuzhou, 350002 China; 4 Fujian University Engineering Research Center for Modern Agricultural Equipment, Fuzhou, 350002 China

**Keywords:** Hyperspectral imaging, Internal attribute evaluation, Carrot, Variable selection, Quantitative analysis model, Biochemistry, Biological techniques, Plant sciences

## Abstract

The study aimed to measure the carotenoid (Car) and pH contents of carrots using hyperspectral imaging. A total of 300 images were collected using a hyperspectral imaging system, covering 472 wavebands from 400 to 1000 nm. Regions of interest (ROIs) were defined to extract average spectra from the hyperspectral images (HIS). We developed two models: least squares support vector machine (LS-SVM) and partial least squares regression (PLSR) to establish a quantitative analysis between the pigment amounts and spectra. The spectra and pigment contents were predicted and correlated using these models. The selection of EWs for modeling was done using the Successive Projections Algorithm (SPA), regression coefficients (RC) from PLSR models, and LS-SVM. The results demonstrated that hyperspectral imaging could effectively evaluate the internal attributes of carrot cortex and xylem. Moreover, these models accurately predicted the Car and pH contents of the carrot parts. This study provides a valuable approach for variable selection and modeling in hyperspectral imaging studies of carrots.

## Introduction

Carrot (*Daucus carota* L.) is a widely consumed root vegetable crop known for its high nutritional value, including essential micronutrients such as vitamins A and C^1,2^. Carrot production is rising worldwide, with China leading the way as the top producer^3^. Although carrots are typically orange, they also exhibit a range of other colors including purple, red, and yellow, thereby enriching the diversity within the spectrum^4^. Moreover, these crops provide significant amounts of antioxidants, provitamin A, and carotenoids, which have been linked to various health benefits, including a lower risk of prostate cancer and improved heart and liver health^5–8^.

With its unique pH value, carrot juice is susceptible to spoilage and pathogenic organisms^9^. Key quality indicators for carrots include factors like color, absence of bruises, provitamin A content, vitamin C levels, and firmness, all of which impact shelf life, market value, and consumer satisfaction^10^. Carrots' shelf life, selling price, and customer satisfaction depend on their quality. Enhancing carrot quality inspection and developing rapid quality control technologies that give precise and detailed information about nutritional content is crucial, given rising consumption and the effects of climate change^11,12^. This information can be utilized to ascertain the most suitable time for harvesting, refine storage parameters, and enhance the nutritional quality of processed carrot derivatives.

The simultaneous collection of spectral and image data from the tested sample using hyperspectral imaging (HSI) merges conventional spectroscopy and digital imaging technology into a system^13–15^. HSI technology is used in various industries, including agriculture, food^16^, environmental management, and urban planning. It can provide substantial information in spectral and spatial domains^17^. In recent years, HSI technology has played a pivotal role in detecting the internal quality of agricultural products, ranging from moisture and starch detection contents^18^ to protein and fat analysis. Furthermore, HSI has also been leveraged to investigate crop diseases^19^, nutrient deficiency^20^, and estimating biochemical and biophysical characteristics essential for understanding vegetable physiological status and predicting crop yields. Moreover, this tool can investigate soil properties, including moisture content, organic matter, and carbon content^21,22^, total capsaicinoids^23^, and pH^24^. Munera et al.^25,26^ mentioned that the evaluation of fruit quality is a recently developed application. For instance, studies on the quality detection of bakery goods, meat, and fresh vegetables have already been published^27^.

**Figure 1:**
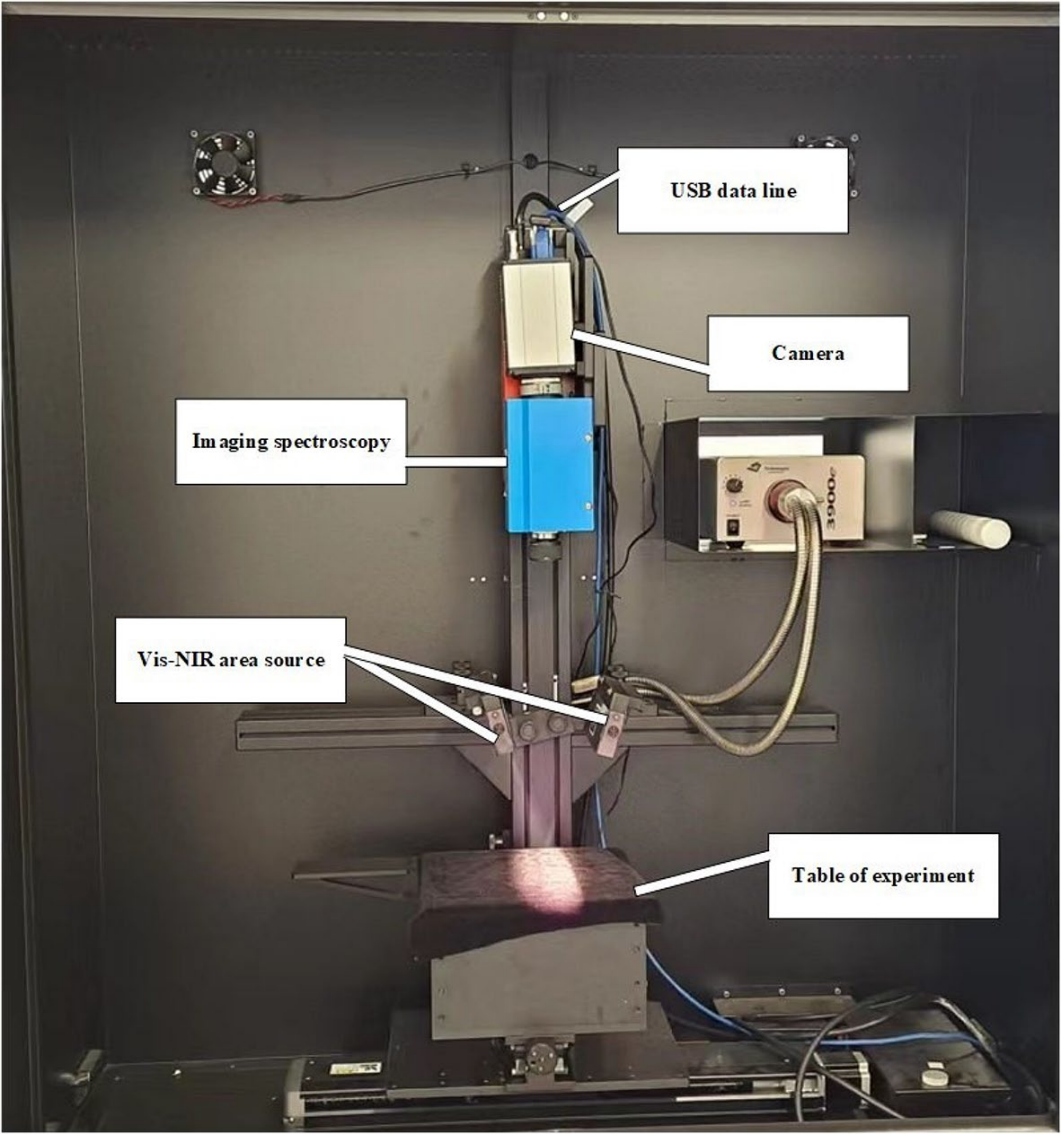
Hyperspectral imaging system.

Research has shown that visible/near-infrared HSI technology has been extensively employed in the non-destructive assessment of interior fruit attributes, including soluble solids content (SSC) and firmness. Nevertheless, the current research on predicting Car and pH content in various regions of fruits, such as the cortex and xylem, is limited from a scientific standpoint. To comprehensively evaluate the internal quality attributes of carrots, this study aimed to investigate the potential of hyperspectral reflectance imaging for predicting the Car and pH content of carrots. We sought to investigate these parameters in two central regions of carrots (cortex and xylem) using visible and near-infrared (Vis/NIR) HSI. The specific objectives of this study were to:

1. Acquire hyperspectral images of carrot samples and extract spectral data from them.
2. Build partial least squares regression (PLSR) and least squares support vector machine (LS-SVM) models using the entire spectrum.
3. Choose representative wavelengths using successful projection algorithms (SPA) and regression coefficients (RC) from PLSR.
4. Develop simplified LS-SVM and PLSR models.
5. Use the best model to predict the quality attributes of each sample pixel and compare its performance to Fig. 1.

## Methods

### Sample preparation

A stratified sampling approach was applied to select carrot samples for analysis. A comprehensive collection of 300 carrot samples, exhibiting comparable shape and size, was procured from Putian (Pt) and Fuzhou (Fz) City, located within the geographical boundaries of Fujian Province, China. Each sample weighted between 55 to 65 g.

Following an exhaustive washing process, carrots that exhibited cracks, rust, dysmorphia, or dark discoloration were excluded from the sample set. As a result, 240 samples remained, all meeting the predefined quality criteria. Among the samples selected for investigation, 120 carrots were sourced from the Fz, while the remaining 120 originated from the Pt. The carrots were stored in a sealed plastic bag at 3 °C for 2 days. Later, each carrot was divided into two halves to investigate Car and pH contents. The plant material used in this work complies with relevant institutional, national, and international guidelines and legislation.

### HSI system and image acquisition

Experimental HSI was conducted using a high-performance CCD digital camera (Sencicam QE Taiwan) and a hyperspectral camera (HIS-V10E-sCMOS) that covered the wavelength range of 400–1000 nm with a spectral resolution of 2–8 nm. The system was equipped with Oriel Instruments USA halogen tungsten light bulbs, a spatial resolution point radius of 9 m, a light source supply system with a feedback controller, and a computer. The camera was operated using Camera Control Kit V219. We used this camera to capture hyperspectral images. The camera consisted of a focal length of 170mm, a scanning line distance of 2mm, and a light source beam's optical center located 2mm from the scanning line. We integrated data from four evenly spaced places over the equator using a 22-binning technique to provide a full spectral image to conduct the HSI of the carrots. See Fig. 1 for a graphic depiction of the HSI equipment.

### Image processing

**Figure 2:**
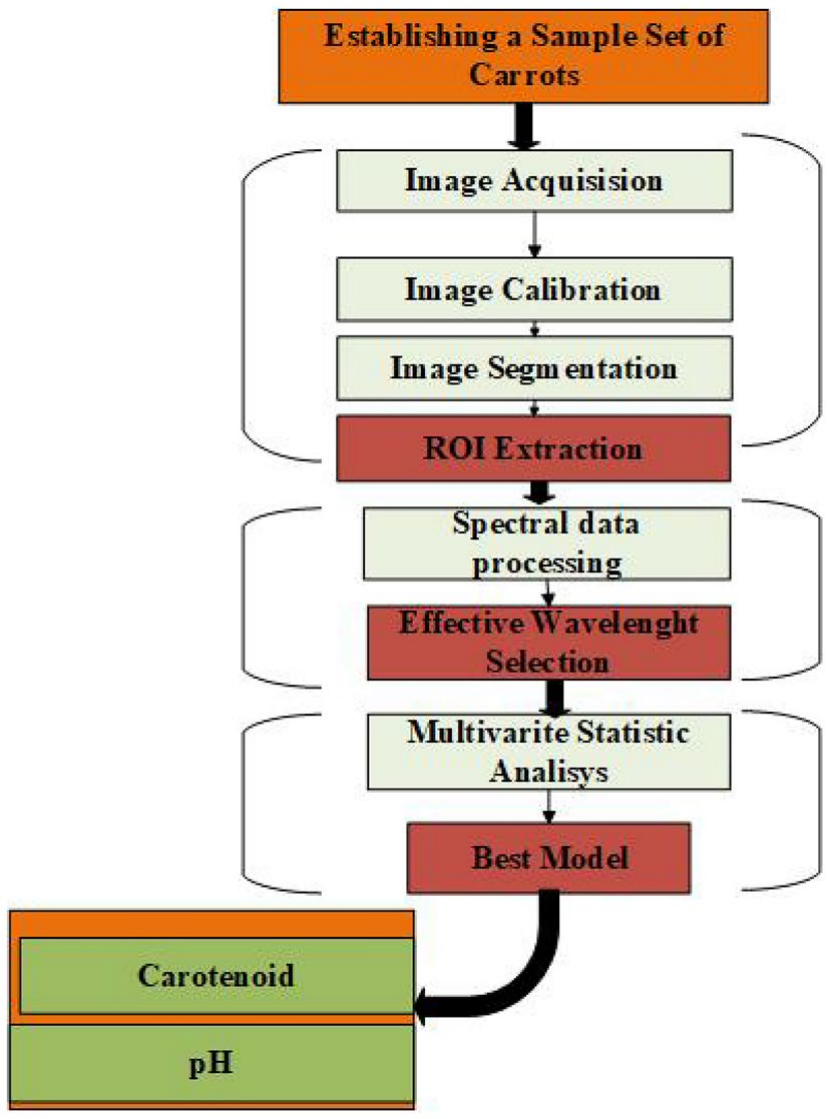
Flow diagram of the experimental steps.

One of the most important steps in pre-processing the hyperspectral images was calibrating the raw data to exclude dark current effects from the CCD camera. After calibration, an area of interest (ROI) was found in the calibrated images, and spectrum data was then taken out of these ROIs, as Fig. 2 shows. To reduce differences caused by illumination, detector sensitivity, camera specs, and subtleties in the physical setup, raw hyperspectral photos were corrected by comparing them to black-and-white reference images^28^.

The camera lens was covered with its opaque cap, and the light source was turned off to provide a black reference image. Alternatively, a spectral image of a uniformly white tile with approximately 99.9% reflectance was captured to create a white reference image^29^. The following equation was used to adjust the uncorrected hyperspectral pictures:

1 $$R=\frac{I-{I}_{d}}{{I}_{w}-{I}_{d}}$$

Here, R represents the corrected hyperspectral image, while $I$ represents the sample's initial spectral image, ${I}_{d}$ denotes the dark reference image and ${I}_{w}$ standsfor the white reference image. We leveraged image acquisition software to correct the image.

### Spectral pre-treatment

The hyperspectral data were extracted from the acquired ROIs for spectral processing. Undesired variations were compensated (negative effects from random and systematic noise), and unnecessary or noisy wavelengths were removed to improve prediction accuracy. Pre-treatment was applied to the spectral data in the form of operations, including smoothing, derivatives, multiplicative scatter correction (MSC)^14,15^, standard normal variate (SNV), and Savitzky–Golay (SG)^30^. The SG smoothing method with a window width of three points was used to reduce high-frequency noise, baseline excursion, and dispersion to stabilize the baseline and reduce noise. Moreover, MSC was applied to adjust for additive and multiplicative scatter effects, which improved and corrected the obtained hyperspectral data.

The SG filter^31^ is a widely used technique for smoothing data based on approximating the raw data using polynomials in a defined data frame. The SG filter has two degrees of freedom, including polynomial order and window length. The first parameter enables the smoothed data to follow the raw data as closely as possible. This process demonstrates the importance of preserving the edges of the data. However, it also entails the drawback of tracking noise fluctuations. The window length neutralizes the high-frequency noise contribution for the second degree of freedom by smoothing its fluctuations through polynomial fitting^32,33^. The SG filter searches for the optimal n + 1 polynomial coefficients for a given n-degree polynomial to best suit the raw data and assesses the outcome in the window center^34,35^. The polynomial function was applied to the signal point by point. The measured value of the window's midpoint was replaced with the polynomial function's estimated value. The degree of smoothing was altered by changing the window's width and polynomial order. In addition to SG smoothing, other spectral pre-treatment methods, such as MSC and SNV, are commonly used to compensate for undesired variations and remove unnecessary or noisy wavelengths^14,15,30^.

### Effective wavelength selection methods

The spectrum data set may comprise thousands of variables/wavelengths and hundreds or thousands of samples^36,37^ due to the high resolution of modern spectroscopic instruments. Such large-scale data can make hyperspectral image inspection techniques more time-consuming. Moreover, variable selection (wavelength selection) is crucial in identifying the relevant variables and eliminating highly correlated ones to reduce computational complexity, increase detection effectiveness, and meet the industry-required inspection speed^38,39^. While no definitive method has been established for selecting optimal wavelengths, various approaches have been recommended^40^. For instance, SPA, RC, uninformative variable elimination (UVE), simulated annealing (SA), K-nearest neighbors regression (K-NNR), and genetic algorithm (GA) are a few multivariate algorithms that have been suggested for developing quantitative models.

In this study, the wavelength selection techniques utilized included RC, K-NNR, and SPA. SPA identified wavelengths with the least redundant information. SPA has been described as a method for identifying relevant features in a forward direction by comparing projection vectors resulting from projecting wavelengths onto other wavelengths. It chooses the most significant projection vector wavelength and incorporates it into the candidate subset of characteristic wavelengths. Studies have described SPA as a method that identifies relevant features in a forward direction by comparing projection vectors resulting from projecting wavelengths onto other wavelengths. It selects the most significant projection vector wavelength and includes it in the candidate subset of characteristic wavelengths^41^. Here, the performance of different subsets was evaluated using a regression model. SPA aims to identify a combination of variables that contains the least redundant information and the least covariance, thereby reducing model complexity and improving accuracy. Overall, SPA is a useful tool for feature selection in various applications, such as regression, classification, and data mining.

It has been established that RC plays a decisive role in creating a predictive model for specific data collection. Weighted RC, also known as b-coefficients, which are equivalent to the model with full spectra, are used to calculate RC. The best wavelengths are determined by selecting those with the highest absolute b-coefficient values. This approach enables the identification of the most crucial wavelengths for forecasting the response variable, leading to a more accurate and effective model^42^. The use of fewer wavelengths in spectral analysis has the potential to improve model performance^24,24^. The method representing a small number of wavelengths, RC, and SPA, was chosen for modeling following the selection of EWs.

### Modeling methods and model evaluation

Model validation is an important step in multivariate data analysis. The prediction model for this study was constructed utilizing PLSR and LS-SVM, which are linear multivariate algorithms. This is because of its efficacy when a linear relationship exists between spectra and object properties^43–45^. PLSR is widely employed in chemometrics to analyze the correlation between spectral data and reference quality indicators. A set of statistically uncorrelated latent variables was utilized by the PLSR model to forecast Car and pH levels. Through decomposition, this method generates principal factors from the independent and dependent variables as they are projected into a new multidimensional space. Seven PLSR factors were chosen for this investigation according to the correlation strength of the principal factors. Notably, PLSR and LS-SVM were applied to the prediction model. However, PLSR was solely utilized to model the full spectra^44^.

Based on concepts from statistical learning theory, SVM can be used for classification and nonlinear regression. LS-SVM is an enhancement of traditional SVM. It uses least-squares linear systems as the loss function rather than traditional convex quadratic programming^46^. LS-SVM is more than SVM because of its low computational complexity and efficiency.

2 $$y\left(x\right)={\sum }_{k=1}^{N}{a}_{k}K\left(x,{x}_{k}\right)+b$$

In the given context, $K\left(x,{x}_{k}\right)$ represents the kernel function, ${x}_{k}$ indicates the input vectors, *k* denotes the support values, and $b$ indicates the bias factor. The computation of similarity between the input vectors is the responsibility of the kernel function, and the kernel function selection influences the efficacy of the model.

Furthermore, a correlation analysis was conducted to assess the RC of the simplified models, investigating the association between the EWs and the quality features.

#### Model evaluation

The prediction capacities of the models were assessed by calculating statistical metrics, including the coefficient of determination of calibration (R^2^_cal_), coefficient of determination of prediction (R^2^_pre_), root mean square error (RMSEC, RMSEP), and RPD can be described as follows:

3 $${R}^{2}=\frac{{{\sum }_{i}(\widehat{y}}_{i}-{y}_{i}{)}^{2}}{{\sum }_{i}(\overline{y }-{y}_{i}{)}^{2}}$$

4 $${\text{RMSE}}=\sqrt{\frac{1}{{\text{m}}}{\sum }_{{\text{i}}=1}^{{\text{m}}}(\widehat{y}-{y}_{i}{)}^{2}}$$

5 $${\text{RPD}}=\frac{{\text{SD}}}{{\text{RMSE}}}$$

where m represents the number of samples, ${\widehat{{\text{y}}}}_{{\text{i}}}$ represents the predicted value, ${{\text{y}}}_{{\text{i}}}$ represents the actual value and $\overline{{\text{y}} }$ represents the mean value of the actual value. SD is the standard deviation of the validation sample.

When the RPD value is greater than 2.5, it indicates a high capacity for prediction^47^. Spectral data extraction was conducted on ENVI 4.8 (ITT, Visual Information Solutions, Boulder, USA). All computations and multivariate data analyses were performed with chemometric software Unscrambler^®^ 9.7 (CAMO AS, Oslo, Norway) and MATLAB R 2009b (The Math Works, Natick, USA).

### Biochemical analyses

After acquiring hyperspectral images, the samples were immediately sliced and weighed for subsequent chemical analysis. Each measurement was performed three times^48^. We used 0.1 g of fresh-weight material immersed in a 20 ml solution containing 80% acetone and 100% ethanol (1:1 ratio) for 24 h in darkness to extract the pigments. The pH composite electrode was mixed in pure water and then shaken dry after being thoroughly washed. The pH meter was placed into the 4.00 pH calibration solution to calibrate it. Once the calibration was finished, the meter was rinsed with distilled water and dried. The pH meter was calibrated using a standard buffer solution with a pH of 7.00^49,50^. The meter was cleaned with pure water, dried, and calibrated using a pH 9.18 solution. The three-point calibration has been accomplished at this stage. A sufficient amount of pulp was extracted from each sample, squeezed to obtain juice, and then the electrode was immersed in the juice to measure the pH value. Next, the electrode was immersed in the juice, and the pH value was measured. Each sample underwent three measurements following the described procedure. The average of the three readings was considered for the pH value.

We measured the Car levels with a 752UV/Vis spectrophotometer and determined based on fresh weight using standard techniques^51,52^.

## Results

### Hyperspectral reflectance spectra

**Figure 3:**
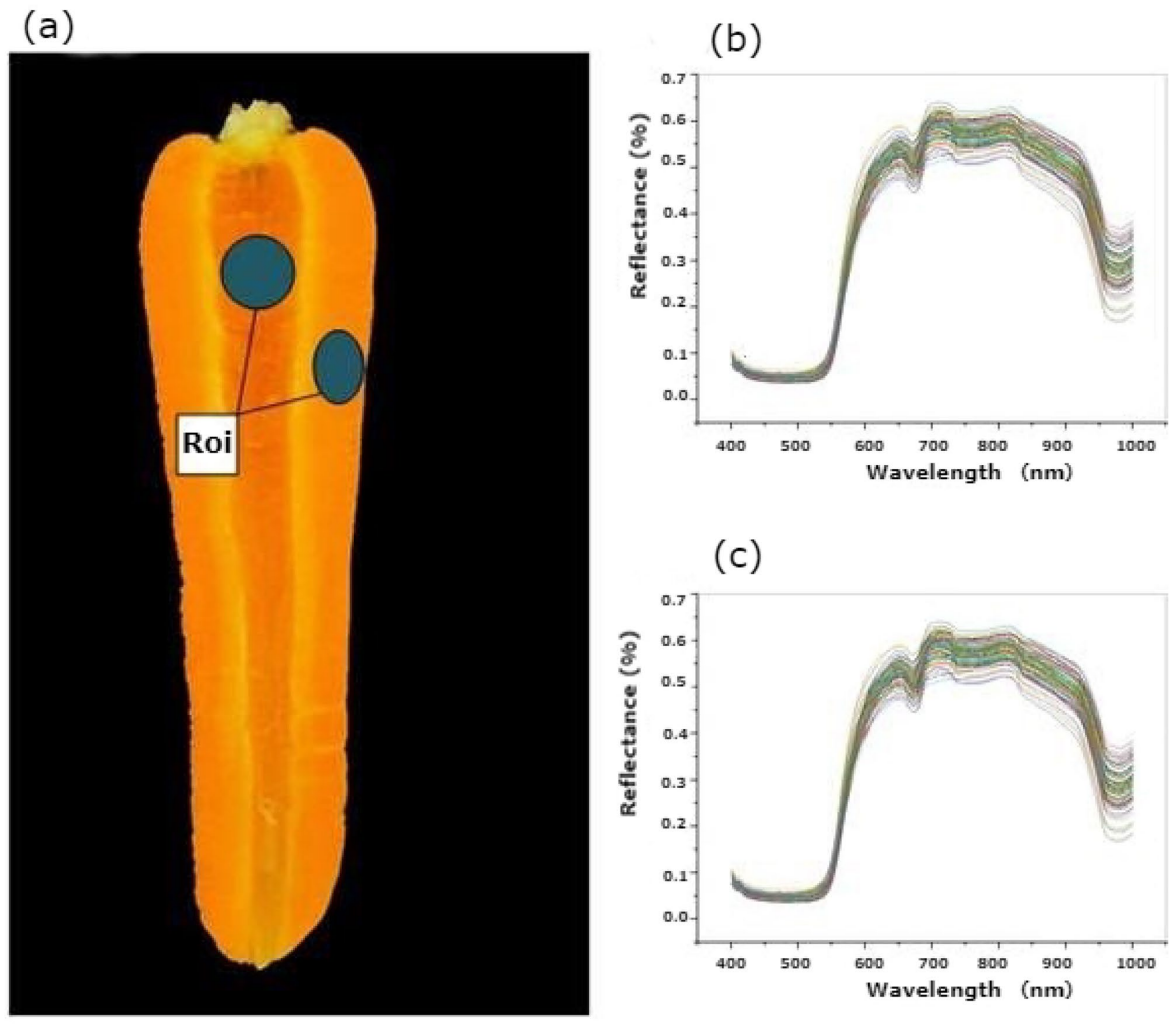
Main steps for image and spectra processing of carrot: (**a**) identification of the Region of Interest (ROI), (**b**) Fz-cortex raw mean reflectance spectrum, and (**c**) Fz-xylem raw mean reflectance spectrum.

**Figure 4:**
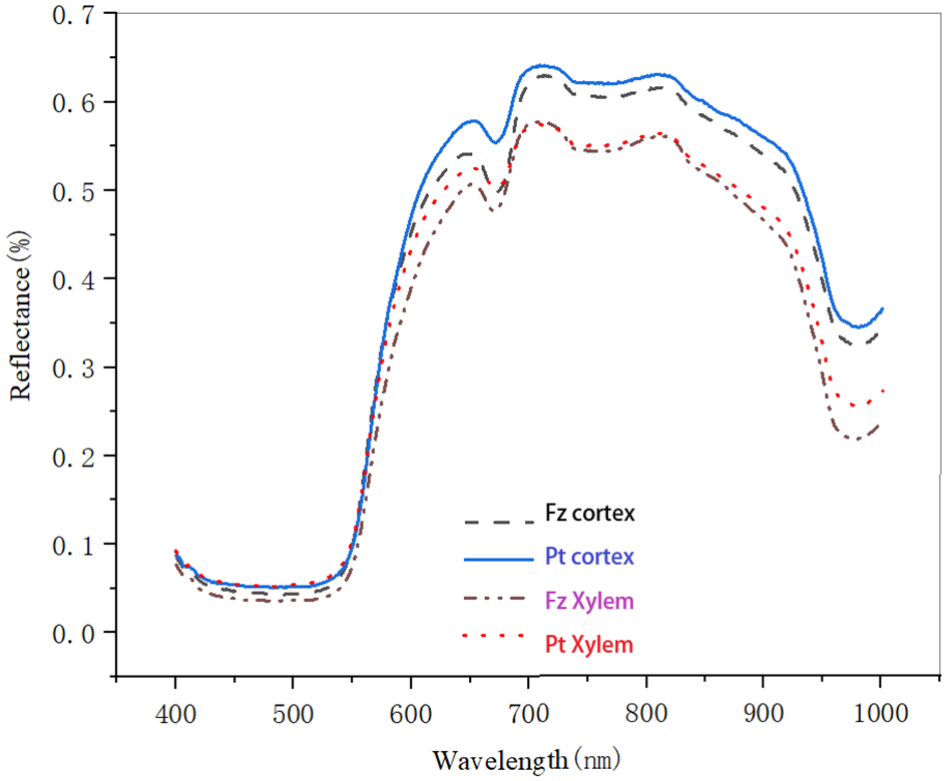
Average spectral curves of cortex and xylem of the Fz and Pt carrot cultivar.

Figure 3a shows how to identify the carrot region of interest. Figure 3b and c show the 400–1000 nm xylem and cortex spectral of the Fz carrot cultivar, respectively. These spectra were taken from the hyperspectral image of calibration set samples. It is evident that the spectra from all sides follow the same pattern across the whole wavelength range, but there were some notable deviations. The spectral curves exhibited distinct absorption and reflection peaks, as can be seen in Fig. 4. The reflectivity of the Fz-xylem and Pt-xylem side is slightly higher than that of the Fz-cortex and Pt-cortex side within the visible light range of 420 to 680 nm, which was based on the spectral images obtained from the Fz-xylem and Pt-xylem, as well as the Fz-cortex and Pt-cortex side. However, the reflectivity of the Fz-xylem and Pt-xylem side significantly increases compared to the Fz-cortex and Pt-cortex side within the near-infrared range of 780 to 1000 nm.

A typical Car absorption band at 680nm corresponds to the first discernible absorption peak. Around 750 nm is the peak of the second absorption center, and a relatively wide absorption band is connected to the band C–H's fourth overtone. The second overtone of band O–H may be related to the tiny absorption band at 950 nm^53^.

In addition to the typical absorption characteristics, the spectral intensities of different samples were different, indicating differences in chemical components, which was conducive to constructing the Car and pH quantitative analysis model.

### PLSR models based on the full spectra

We leveraged PLSR to establish regression models with the xylem and cortex datasets. The regression results are shown in Tables 1 and 2. PLSR models, the samples were taken in the same order on the carrot xylem and cortex side. The calibration and prediction sets for both regions of the carrot were also the same.

**Table 1:** Results and parameters of the calibration and prediction sets of Car by partial least squares regression (PLSR) models.

Cultivar	Region	Calibration set		Prediction set		
		R^2^_Pre_	RMSEC	R^2^_Pre_	RMSEP	RPD
Fz	Xylem	0.924	0.021	0.903	0.026	2.19
Fz	Cortex	0.897	0.023	0.885	0.025	1.61
Pt	Xylem	0.930	0.022	0.915	0.027	2.21
Pt	Cortex	0.906	0.025	0.876	0.030	1.72

**Table 2:** Results and parameters of the calibration and prediction sets of pH by PLSRmodels.

Cultivar	Region	Calibration set		Prediction set		
		R^2^_Pre_	RMSEC	R^2^_Pre_	RMSEP	RPD
Fz	Xylem	0.799	0.024	0.666	0.022	1.54
Fz	Cortex	0.841	0.024	0.682	0.025	1.72
Pt	Xylem	0.854	0.023	0.674	0.027	1.66
Pt	Cortex	0.871	0.024	0.702	0.035	1.80

As indicated by the R^2^_pre_, RMSEP, and RDP values, the results displayed in Table 1 illustrate the ability to predict carotenoid quality and pH in the xylem and bark regions of the Fz-Pt cultivar. The RMSEP values for Fz and Pt in the xylem region were 0.026 and 0.027, respectively, while the R^2^_pre_ values for predicting carotenoid quality ranged from 0.903 to 0.915 for Fz and from 0.885 to 0.876 for Pt. Furthermore, the region where Fz and Pt had RDP values of 2.19 and 2.21, respectively, demonstrated a greater ability to predict carotenoid quality. In contrast, the R^2^_pre_ values obtained to infer pH in the xylem and cortex regions were comparatively lower, ranging from 0.666 to 0.702. Furthermore, RMSEP values ranged from 0.022 to 0.035. All RDP values were less than 2, indicating a satisfactory level of predictive accuracy despite the lower R^2^_pre_ values. It was also observed that the RDP values in the cortex region were slightly higher than those in the xylem region. This discrepancy implies that pH prediction performance was significantly improved in the cortex region.

### Selection of effective wavelengths

Choosing a configuration with fewer wavebands is recommended to enhance the stability and integrability of a multispectral imaging system from a scientific standpoint (ElMasry et al. 2019^54^). SPA was used to identify the EWs carrying crucial information for determining scaling rates and reducing data dimensionality. These EWs remove unnecessary information by including the whole spectral data range (400–1000 nm), representing the most important data among the EWs. Table 3 demonstrates that only the important wavelengths are required to estimate Car and pH. The pH decreased the number of wavelengths from 5 to 9, contrasting the pH range (8 to 14) detected in the xylem of both cultivars, which showed a wavelength range from (Table 3).

**Table 3:** The selected EWs for Car and pH using RC and SPA.

Cultivar	Parameter	Selected range (nm)	Methods	Region	Number	Selected EWs (nm)
Fz	Car	400–1000	RC	Xylem	10	910, 643, 441, 561, 628, 880, 664, 731, 450, 945
				Cortex	7	410, 580, 671, 900, 950, 660, 730
Pt	Car	400–1000	RC	Xylem	14	994, 444, 561, 594, 628, 880, 577, 634, 658, 660, 730, 739, 740, 765
				Cortex	10	775, 410, 580, 670, 952, 950, 662, 685, 704, 736
Fz	Car	400–1000	SPA	Xylem	11	902, 440, 560, 681, 594, 625, 880, 958, 956, 828, 691
				Cortex	8	489, 522, 555, 584, 582, 671, 911, 953
Pt	Car	400–1000	SPA	Xylem	14	994, 574, 444, 561, 580, 628, 881, 577, 634, 659, 662, 730, 739, 765
				Cortex	13	543, 595, 674, 775, 410, 673, 952, 952, 662, 685, 699, 704, 736
Fz	pH	400–1000	RC	Xylem	8	441, 561, 594, 628, 880, 994, 674
				Cortex	7	410, 580, 671, 900, 950, 704, 735
Pt	pH	400–1000	SPA	Xylem	8	441, 561, 594, 628, 880, 994
				Cortex	5	410, 580, 671, 900, 950
Pt	pH	400–1000	SPA	Xylem	8	443, 556, 592, 628, 880, 992, 674, 710
				Cortex	9	410, 714, 721, 580, 673, 900, 950, 702, 734

#### Prediction of pH

Table 3 illustrated that the xylem spectra had less spectral than the cortex spectra. However, their predictive capability was much better.

Besides, Table 4 showed that the prediction under the RC-PLSR model had an R^2^_Pre_ of 0.672 and an RMSEP of 0.030, while the counterpart had an R^2^_Pre_ of 0.752 and an RMSEP of 0.029. However, the RC-LS-SVM model had an R^2^_Pre_ of 0.701 and an RMSEP of 0.032, while the counterpart had an R^2^_Pre_ of 0.802, an RMSEP of 0.026, an R^2^_Pre_ of 0.757, and an RMSEP of 0.024.

**Table 4:** LS-SVM and PLSR models calibration and prediction of pH using EWs (RC and SPA).

Cultivar	Models	Region	Calibration set		Prediction set		
			R^2^_Pre_	RMSEC	R^2^_Pre_	RMSEP	RPD
Fz	RC-PLSR	Xylem	0.901	0.025	0.672	0.030	1.67
Fz	RC-PLSR	Cortex	0.961	0.021	0.752	0.029	1.72
Pt	RC-PLSR	Xylem	0.892	0.026	0.653	0.029	1.55
Pt	RC-PLSR	Cortex	0.924	0.022	0.711	0.030	1.70
Fz	RC-LS-SVM	Xylem	0.876	0.024	0.701	0.032	1.56
Fz	RC-LS-SVM	Cortex	0.957	0.030	0.802	0.026	1.92
Pt	RC-LS-SVM	Xylem	0.861	0.023	0.696	0.030	1.57
Pt	RC-LS-SVM	Cortex	0.946	0.030	0.812	0.025	2.00
Fz	SPA-PLSR	Xylem	0.923	0.027	0.678	0.031	1.61
Fz	SPA-PLSR	Cortex	0.964	0.024	0.841	0.024	2.08
Pt	SPA-PLSR	Xylem	0.933	0.025	0.681	0.031	1.62
Pt	SPA-PLSR	Cortex	0.953	0.024	0.791	0.025	2.11
Fz	SPA-LS-SVM	Xylem	0.826	0.025	0.731	0.030	1.67
Fz	SPA-LS-SVM	Cortex	0.954	0.023	0.816	0.028	1.79
Pt	SPA-LS-SVM	Xylem	0.841	0.024	0.742	0.029	1.81
Pt	SPA-LS-SVM	Cortex	0.970	0.024	0.820	0.026	2.40

The SPA-PLSR had an R^2^_Pre_ of 0.678 and an RMSEP of 0.031, while the SPA-LS-SVM had an R^2^_Pre_ of 0.731 and an RMSEP of 0.030, and the counterpart had an R^2^_Pre_ of 0.816 and an RMSEP of 0.028. The identical outcome observed in the Fz sample was found to be applicable in the Pt samples, as evidenced by the data presented in Table 4. Our findings indicate that the spectral characteristics of the xylem were more effective in predicting Car than those of the cortex.

#### Prediction of carotenoid

Here, we used EWs to build Car and pH-predicting models in two carrot cultivars, namely Fz and Pt. The obtained results of the car prediction are shown for the Fz sample in Table 5.

**Table 5:** LS-SVM and PLSR models calibration and prediction of pH using EWs (RC and SPA).

Cultivar	Models	Region	Calibration set		Prediction set		
			R^2^_Pre_	RMSEC	R^2^_Pre_	RMSEP	RPD
Fz	RC-PLSR	Xylem	0.937	0.022	0.892	0.023	2.17
Fz	RC-PLSR	Cortex	0.906	0.025	0.854	0.030	1.67
Pt	RC-PLSR	Xylem	0.941	0.021	0.890	0.024	2.16
Pt	RC-PLSR	Cortex	0.961	0.024	0.872	0.029	2.00
Fz	RC-LS-SVM	Xylem	0.961	0.023	0.933	0.022	2.27
Fz	RC-LS-SVM	Cortex	0.914	0.024	0.883	0.026	1.82
Pt	RC-LS-SVM	Xylem	0.957	0.024	0.878	0.022	2.13
Pt	RC-LS-SVM	Cortex	0.930	0.023	0.842	0.026	1.57
Fz	SPA-PLSR	Xylem	0.933	0.022	0.896	0.023	2.08
Fz	SPA-PLSR	Cortex	0.913	0.024	0.815	0.024	2.08
Pt	SPA-PLSR	Xylem	0.923	0.022	0.944	0.024	2.27
Pt	SPA-PLSR	Cortex	0.933	0.023	0.811	0.022	1.76
Fz	SPA-LS-SVM	Xylem	0.967	0.025	0.934	0.022	2.30
Fz	SPA-LS-SVM	Cortex	0.922	0.022	0.893	0.024	2.17
Pt	SPA-LS-SVM	Xylem	0.964	0.024	0.932	0.022	2.26
Pt	SPA-LS-SVM	Cortex	0.924	0.021	0.831	0.023	1.80

Table 5 also showed that the Fz cultivar RC-PLSR model had an R^2^_Pre_ value of 0.892 and an RMSEP value of 0.023 in the xylem, slightly lower than its counterpart (using the cortex), with R^2^_Pre_ = 0.854and RMSEP = 0.030. The RC-LS-SVM results showed an R^2^_Pre_ value of 0.933, an RMSEP value of 0.022, and an RPD of 2.27, while its counterpart had an R^2^_Pre_ value of 0.883 and an RMSEP value of 0.026, and an RPD of 1.8

For the SPA-PLSR model, the R^2^_Pre_value was 0.896, the RMSEP value was 0.023, and an RDP of 2.17, while its counterpart had an R^2^_Pre_ value of 0.815 and an RMSEP value of 0.024 and an RPD of 2.08. On the other hand, the LS-SVM model had an R^2^_Pre_ value of 0.934 and an RMSEP value of 0.022, with an RPD of 2.27, and its counterpart had an R^2^_Pre_ value of 0.893 and an RMSEP value of 0.024 and 2.08 for the RPD. Regarding prediction accuracy for Car content in both the calibration and prediction sets, the outcomes demonstrate that the LS-SVM models exhibited superior performance overall than the PLSR models. In contrast to the PLSR models, the LS-SVM models demonstrated superior RMSEC and RMSEP values.

The LS-SVM model exhibited notably robust outcomes for the Xylem region among the cultivars, whereas Fz maintained a consistently high performance across all models and regions. Conversely, the cultivar Pt obtained superior performance from the PLSR model in the Cortex region. As listed in Table 5, the Pt samples exhibited the identical pattern identified in the Fz sample. Superior suitability for Car prediction was observed in the xylem spectra compared with the cortex.

**Figure 5:**
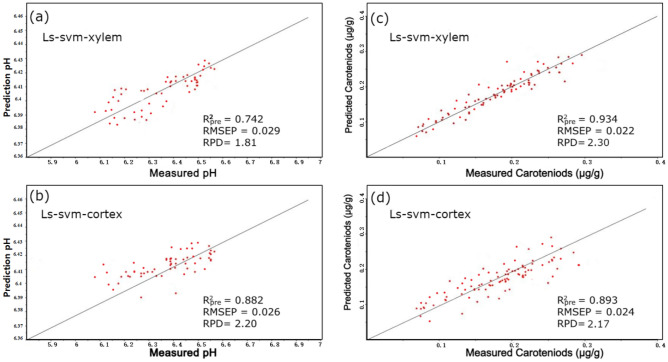
The performances of the top prediction models to detect the quality of Fuzhou (Fz) carrots using EWs are as follows: the pH and carotenoid (Car) prediction is achieved by employing the combination of successive projections algorithm, and least squares support vector machine (SPA-LS-SVM) model.

**Figure 6:**
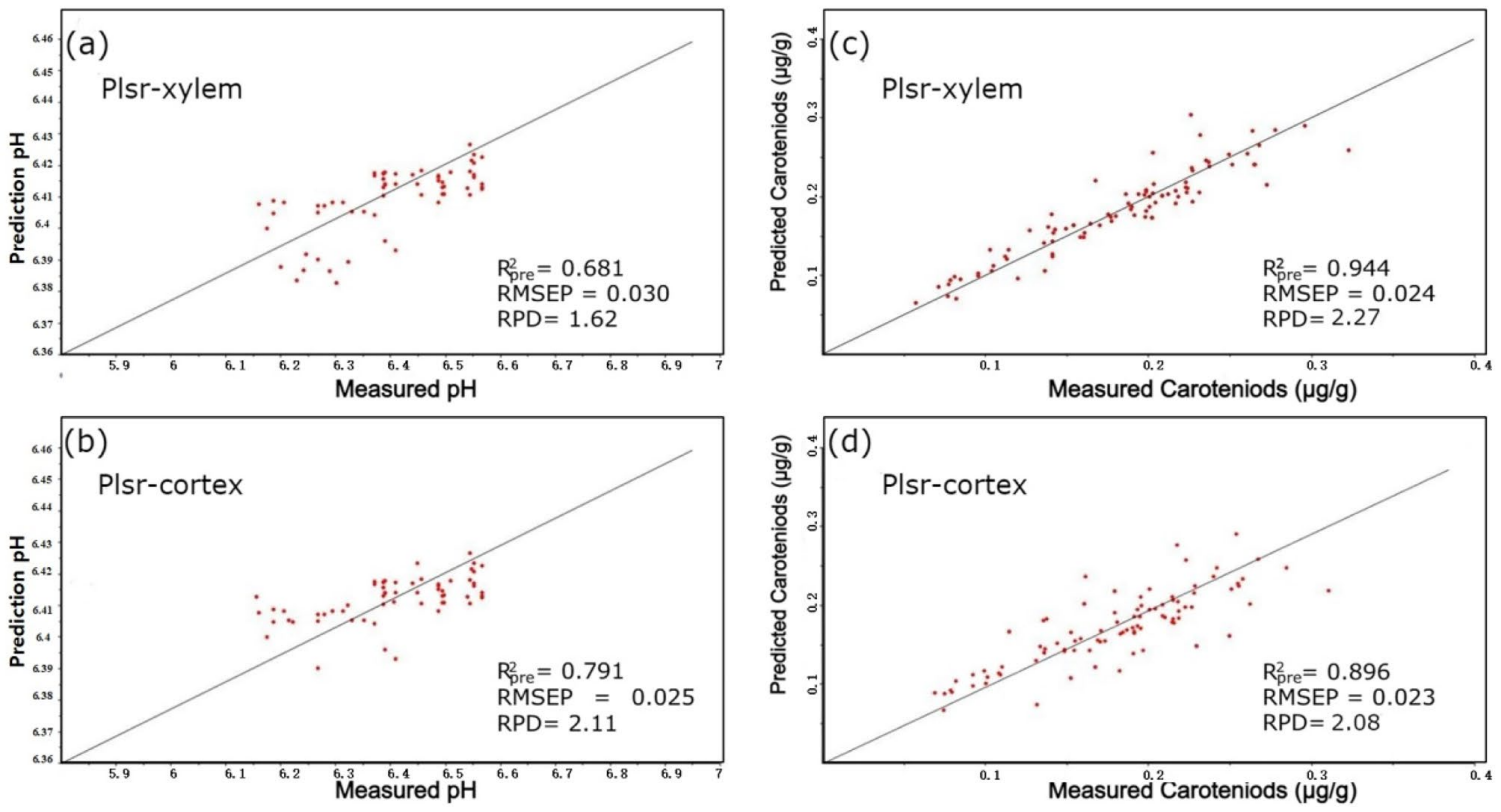
The performances of the top prediction models on Fuzhou (Fz) for detecting quality attributes based on EWs are as follows: the pH and caroteniod (Car) prediction is achieved by employing the combination of successive projections algorithm and partial least squares regression (SPA-PLSR) model.

Figures 5 and 6 illustrate the optimal prediction outcomes according to the selected-range spectra.

## Discussion

### Analysis of characteristic wavelengths

Here, the spectral window ranged from 400 to 1000 nm. The outcomes of the wavelength selection are shown in Table 1. The EWs for Car were determined in the xylem and cortex, ranging from 410 and 956 nm, whereas the EWs for pH contents were between 500 and 900 nm. In related research, Car pigments were observed at wavelengths between 400 and 500 nm^55^, 450 nm, and 580 nm^25,26^, as well as 400–600 nm^56^. Additional peaks were observed at the xylem area at 820 and 980 nm and in the cortex region at 814 and 970 nm. Additionally, acids were found to be present at 800 nm^56^, and sugars were detected at 835 nm^24^ and 840 nm^57^. Therefore, the peak at 820 nm in the carrot xylem and 814 nm in the cortex could be related to acids and sugars in both cases. Water was detected at 960 nm^58^, 970 nm^56^, and 976 nm^24^. Hence, the peaks reported at 980 nm and 970 nm may be attributed to water and sugars. Conversely, water and sugars have been observed at wavelengths of 970 nm^59,60^, 960–980 nm^61^, and 970–980 nm^62^, respectively. In particular, some minor differences in wavelength reflectance were observed in the xylem and cortex.

### Selection of effective wavelengths

The research emphasized the criticality of EW selection, improving the stability and integrability of multispectral imaging systems. The study employed SPA and RC to forecast quality metrics associated with Car and pH, as suggested in ElMasry et al. (2019^54^) work for a system with a diminished quantity of wavebands. By the selection procedure, informative EWs containing crucial information for detecting scaling rates were discerned, resulting in the compilation of a more efficient dataset comprising the most valuable spectral data. The 400–1000 nm wavelengths were considered essential for predicting Car and pH characteristics.. Notably, the chosen EWs exhibited variations between the xylem and cortex regions of the cultivars, suggesting that the estimation of Cars requires distinct spectral information needs.

In addition, the analysis unveiled particular wavelength requirements for pH estimation, suggesting the presence of more concentrated and accurate spectral data that is essential for precise pH forecasting. The application of SPA in ascertaining the chosen EWs enhanced the accuracy of the prediction models by revealing the crucial wavelengths associated with each quality attribute and emphasizing the combined benefits of RC and SPA methods. Consistent with previous investigations into internal fruit properties^63,64^, the results of this study provide additional evidence for the importance of vibrational energy changes induced by NIR light on chemical bonds, including C–H, N–H, O–H, and C–O, which influence Near-Infrared Reflectance Spectroscopy^65,66^.

The inconsistency between the NIR method and the number of wavelengths chosen for pH estimation may be attributable to internal pH components that are not perfectly aligned; this highlights the significance of selecting wavelengths tailored to particular attributes^67^.

In general, the predictive capabilities of the models for Car and pH attributes have been improved through the careful selection and analysis of EWs utilizing RC and SPA methods. This highlights the significance of custom wavelength selection in multispectral imaging applications that require accurate and efficient quality attribute predictions in the agricultural and scientific sectors.

### Modeling based on full wavelengths

Tables 1 and 2 show PLSR model carotenoid and pH predictions. Our findings showed that xylem side spectra models outperformed cortex region models. The higher concentration of carotenoids in the xylem area, which transports water and nutrients, may explain this performance differential. Therefore, HSI of xylem side spectra may improve carrot carotenoid predictions.

In contrast, pH prediction performed poorly. Fz-Pt xylem regions had prediction quality of 0.666 to 0.674, while cortical regions had 0.674 to 0.702. Chemical composition and physiological mechanisms may explain the pH prediction performance differential between the xylem and cortex. Plant xylem, which transports water, may have a more stable pH than the cortex, which stores and performs other functions^68,69^. This pH stability variance may explain the decreased xylem forecast accuracy. Even if all RDP values are below 2, the pH prediction models need more development and optimization.

The PLSR models made essentially identical predictions, proving their reliability and consistency.

### Modeling based on effective wavelengths

This study's reference indices for model evaluation were the root mean square error, R^2^_pre,_ and RPD, as shown in Table 3. The regression equations for RC-PLSR-xylem and SPA-LS-SVM-Xylem exhibited high R^2^_pre_ and RPD values and low RMSE values. This suggests that these two differential orders provide superior predictive performance compared to other examples. In summary, the performance of the SPA-PLSR model was found to be slightly worse compared with the other models. However, the SPA-LS-SVM models exhibited exceptional performance. In general, the early warning signals (EWs) identified by the signal processing algorithm (SPA) exhibited more efficacy compared to those discovered by the rule-based classifier (RC).

We noticed that the SPA-LS-SVM method for pH prediction was more accurate than RC, despite RC having more variables. The findings verified that the chosen EWs and spectral morphological parameters were appropriate for spectral dimension reduction and feature extraction. However, they employed different routines for spectral analysis. The former emphasized the morphological differences and spectra variations in samples with diverse Car and pH, while the latter mainly reflected the reflectance or absorbance values. Based on the Car and pH prediction samples, the absorbance spectra calibrated by SG-MSC based on the EWs proved the best in constructing PLSR and LS-SVM predictive models for Car and pH in carrot samples.

On the other hand, Car estimates outperformed pH predictions due to the low organic acid concentration in fruits. Given that the reference pH values did not span a broad spectrum, it is reasonable to infer that they were suitable for establishing a dependable and precise calibration model. The results of the top prediction models for identifying quality attributes using the various variable selection techniques are shown in Figs. 5 and 6.

#### Prediction of pH

The prediction of pH using the full wavelength range was not satisfactory because it did not yield accurate results. However, EWs exhibited an improved prediction^70^. Despite this improvement, the RPD of the pH prediction could not match or exceed the Car prediction. This discrepancy can be attributed to the relatively low concentration of organic acids in the slip fruits^71^. Here, Car prediction relied on a more distinct spectral signature, which made it easier to detect and quantify. The pH values in the cortex tissue were higher than those in the xylem tissue.

Regarding the levels of Car and pH in the different tissues, it is likely that there could be differences in the distribution of these compounds. It has been pointed out that Car are synthesized and stored in plastids in different concentrations in different tissue types^72^. It is also possible that the metabolism of Car and pH in the different tissues is regulated differently, leading to different Car and pH levels^38,39^. Therefore, it is possible to have higher levels of Car in the xylem and lower levels in the cortex, and viceversa for pH levels. However, more research is needed to confirm this phenomenon.

We noticed that the LS-SVM model effectively evaluated the internal qualities of carrots, specifically the pH level, as shown in Table 4. On the other hand, the PLSR presents a substantial discrepancy between the correction set and the prediction generated. Furthermore, the degree of dispersion in the predictions for the data points is considerable, suggesting that the PLSR model exhibits inadequate accuracy in fitting predictions and stability concerning pH quality, as can be seen in Figs. 5a,b and 6a,b. This model can be applied to detect and assess the pH of carrots, providing valuable theoretical support and serving as a foundation for developing online carrot detection equipment. Future research can focus on exploring additional spectral regions and refining the models to improve pH prediction accuracy and further enhance the overall performance of the models.

Moreover, the implementation of the LS-SVM model for assessing carrot quality holds promise for enhancing the agricultural industry. Accurately assessing the pH level of carrots enables farmers and producers to make informed decisions regarding harvesting, storage, and distribution. This process ultimately enhances the market value of the crop and overall quality. Furthermore, successfully applying the LS-SVM model in carrot quality evaluation highlights its potential for use in other fruits and vegetables. This result aligns with the Shao et al.^73^ findings. These authors demonstrated that LS-SVM models exhibit strong predictive capabilities for internal fruit attributes^74,75^. This discovery opens doors for further research and development in agricultural technology, potentially enhancing quality control processes and overall productivity within the industry.

#### Prediction of carotenoid

Research has extensively studied Car in carrot root systems, including tomatoes and peppers^76,77^. For example, Perrin et al. found various Car in carrots with varying root colors. A related work revealed that the orange plant phloem had more Car than its xylem and was in the carrot roots. Another study also documented that the red genotype phloem and xylem Car were similar^78,79^. The overall results of the Car and pH prediction of the two sides showed significant differences using different regression methods. Further analysis of the characteristic wavelengths for Car and pH showed significant similarities between the two regions. Here, Car was mainly distributed on the xylem side, with higher concentrations in the pericarp region of the carrot slice. This difference may be due to differences in Car synthesis and transport between the two tissues. Perrin et al.^78^ found that the varying expression patterns of the genes involved in Car production in various tissues could account for the variations in the accumulation in various root tissues. This aligns with our finding, implying that the cortex side had much lower Car concentrations compared with the xylem tissue. The LS-SVM model predicted significant set of R^2^_pre_values, smaller RMSE values, greater prediction accuracy, and an RPD of 2.30 for the Car of Fz compared to PLSR prediction models, as shown in Table 2 and Figs. 5c,d, and 6c,d. With the least dispersion, the measured and predicted values of the Car of Fz in the LS-SVM model's calibration and prediction sets are situated on opposite sides of the 45° line. This signifies that the model possesses optimal fitting accuracy and stability.

## Conclusion

This study investigated HSI to determine carrots' internal quality, Car, and pH, focusing on the effects of sampling regions of two cultivars. The results indicated that the cortex or xylem region can accurately predict Car and pH, with a significant difference in prediction performance between the two regions. The characteristic wavelengths for Car and pH prediction using different sampling regions were not identical. This suggests that assessing the cortex and xylem regions could be used to predict these attributes more precisely and cost-effectively. This finding is significant as it provides flexibility in the sampling process and allows a more straight forward implementation of HSI in determining internal quality attributes in carrots. This technique can also help farmers better understand their crop's condition and monitor different attributes depending on their supply chains. Overall, our study provides a reference for implementing multispectral technologies for the internal quality assessment of carrots. Adopting HSI can provide accurate and non-destructive testing of internal quality attributes in carrots, benefiting the food industry and, ultimately, the consumers. Further research is needed to fully understand the potential of these models for predicting pH levels in different fruit tissues and under different environmental conditions. Studies are also required to explore different spectral regions to improve the prediction of internal quality attributes in carrots.

## Data Availability

The data used in this study are available upon request. Please contact Shu he zheng at zsh@fafu.edu.cn for access to the data.
